# The Foreign Journals: Chemistry, Pharmacy, &c.

**Published:** 1841-01

**Authors:** 


### CHEMISTRY, PHARMACY, &c.

### *Preparation of Mercurial Ointment in Six Hours.* By M. Faucher.

Among the various methods that are proposed to facilitate the extinction of mercury in fat, the author proposes the following; by which, in the space of six hours, the globules are rendered invisible, either by the naked eye or with the assistance of a lens. It consists of taking

| | |
|---|---|
| Pork-lard . . . . . | 6 ounces |
| White wax, or (which is better) spermaceti . | 2 „ |

They are melted by a gentle heat, and the liquid mass is poured into a cast-iron mortar over two pounds of pure mercury; they are agitated with a wooden pestle till the globules of mercury disappear, and then the mortar is placed on the hot ashes, or over an oil-lamp, to keep the mixture of the consistence of molasses. In this condition it is to be constantly stirred for the number of hours mentioned.

*Giornale delle Scienze Med. di Torino.* 1839.

---

### *Cholestearine in Morbid Fluids.* By Professor Nasse, of Marburg.

In three cases that have lately occurred to him, the author has found plates of cholestearine in the fluid products of disease.

The first case was that of a man with a large bronchocele, of thirty years' standing, which having produced considerable dyspnœa was punctured. A large quantity of fluid was evacuated, and in a portion of it sent to the author, he discovered at once fine particles of cholestearine. Under the microscope they were found to be perfectly transparent, and very thin quadrilateral tablets of an average of one fiftieth of a line in length, and with well-defined nearly right angles. Besides the cholestearine the fluid contained dark, granular, irregularly-rounded globules, which probably consisted of stearine, some oil-globules, and lymph and blood-globules.

The second case was that of a woman, fifty years old, with a very large ovarian dropsy. The fluid that was removed by tapping was brownish, and, in addition to the usual salts, contained a great deal of albumen. With the microscope, there were found in it a number of globules of oil, blood and lymph, and numerous tablets of cholestearine, which measured for the most part one fiftieth of a line in length. There was no fibrine present in the fluid of either this or the preceding case. Half a year afterwards fluid having again accumulated was again drawn off. It now contained fibrine, irregularly-formed pus-globules, and scattered fat-globules, but no cholestearine, nor were any traces of the latter found in the fluid removed by a still later operation.

In the third case, the fluid was taken from an abscess that had formed in consequence of the irritation of an anchylosed shoulder-joint. It was found to contain imperfectly-formed pus-globules, globules of coagulable lymph, minute drops of oil, flocculi of fibrine, and large tablets of cholestearine exactly like those already described. Ten days after none such could be found in the fluid that continued to be sparingly discharged from the wound.

*Müller's Archiv*, 1840. *Heft* iii., p. 267.

